# Data analysis and uncertainty estimation in supercontinuum laser absorption spectroscopy

**DOI:** 10.1038/s41598-018-28705-2

**Published:** 2018-07-09

**Authors:** Johannes Emmert, Niels Göran Blume, Andreas Dreizler, Steven Wagner

**Affiliations:** 1 Department of Reactive Flows and Diagnostics, Technische Universität Darmstadt, 64287 Darmstadt, Germany; 2 High Temperature Process Diagnostics, Technische Universität Darmstadt, 64287 Darmstadt, Germany

## Abstract

A set of algorithms is presented that facilitates the evaluation of super continuum laser absorption spectroscopy (SCLAS) measurements with respect to temperature, pressure and species concentration without the need for simultaneous background intensity measurements. For this purpose a non-linear model fitting approach is employed. A detailed discussion of the influences on the instrument function of the spectrometer and a method for the *in-situ* determination of the instrument function without additional hardware are given. The evaluation procedure is supplemented by a detailed measurement precision assessment by applying an error propagation through the non-linear model fitting approach. While the algorithms are tailored to SCLAS, they can be transferred to other spectroscopic methods, that similarly require an instrument function. The presented methods are validated using gas cell measurements of methane in the near infrared region at pressures up to 8.7 bar.

## Introduction

Strict regulations of pollutant emissions as well as the economic demand for efficiency necessitate the optimization of combustion systems and other high temperature and pressure processes. Measurement systems that allow for the *in-situ* aquisition of concentration, temperature and pressure data with high temporal resolution and without influencing the process itself, are an important tool in this optimization process. Tunable diode laser absorption spectroscopy (TDLAS) fullfills these requirements^1,2^. But unless a complex laser modification of the standard diode laser setup is used^3,4^, TDLAS is limited to a small spectral range of several cm^−1^ (DFB ~2–3 cm^−1^, VCSEL ~10–20 cm^−1^). Therefore the application of TDLAS in high pressure environments is difficult because of the broad spectral lineshapes due to collisional broadening. On the other hand there are spectrocopic techniques like classic fourier transform spectroscopy (FTS), that cover a broad spectral range with high spectral resolution but lack the time resolution to resolve the progress of chemical reactions or process fluctuations. These disadvantages have partially been reduced in new FTS methods like dual comb spectroscopy^5^ (DCS) which increases the time resolution while also avoiding moving parts in the setup. Nonetheless DCS still requires the sampling of several laser pulses for recovering one spectrum. This limits the theoretically achievable repetition rate. The recently introduced super continuum laser absorption spectroscopy (SCLAS) is theoretically able to measure a whole spectrum in a single laser pulse while still covering a broad spectrum of several 100 wavenumbers^6,7^. The broad spectral range that is covered facilitates the simultaneous detection of several species^6^ as well as the simultaneous determination of pressure and temperature from a single measurement^8^. This supercontinuum laser absorption spectroscopy is based on spectrally broad pulses with a duration in the order of 100 ps. First of all this pulse is filtered to limit its spectral bandwidth to the wavelength region on interest. These filtered pulses can then either be dispersed in time^6,7^ or in space^8^ to resolve their spectral content. This work focuses on the dispersion in time, but the presented method is applicable to the space dispersed variant of SCLAS as well.

To achieve the dispersion in time the initially short pulse is passed through a dispersion compensation module (DCM), which results in a temporally stretched pulse with a duration of several 100 ns. This temporal pulse profile reflects the spectral distribution of the initial SCL pulse. The dispersed pulse then passes through the measurement volume where it gets partially absorbed. This step can also be swapped with the DCM as the results are the same in stationary measurement volumes. In instationary volumes passing the pulse through the measurement volume before the DCM can have advantages in terms of a higher signal to noise ratio as a temporally short pulse avoids additional averaging over transients in the system. Nonetheless the evaluation procedure for both cases is the same.

**Figure 1:**
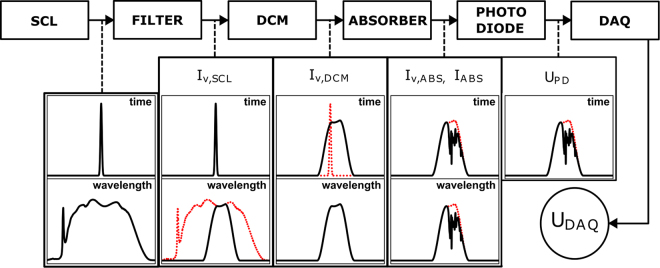
Working principle of a time dispersing SCLAS spectrometer. The initial SCL pulse is spectrally filtered, temporally stretched in a DCM, partially absorbed in the measurement volume and detected on a photo diode before being recorded on an oscilloscope.

Afterwards the intensity profile is detected by a fast photo diode and recorded on a high bandwidth oscilloscope. This measurement principle is summarized in Fig. 1 and is similar to the ones proposed by Orofino *et al*., Kaminski *et al*., Dupont *et al*. and Blume *et al*.^6,7,9,10^. For details we refer to Blume *et al*.^11^, as this work will give a general discussion of the analysis methods for SCLAS measurements.

While a simultaneous reference measurement of the initial intensity distribution simplifies the evaluation procedure, it also introduces disadvantages regarding setup complexity and applicability to harsh industrial environments. E.g. in combustion environments broadband absorbing materials can easily agglomerate or condense on optical measurement accesses. The absorption bands of these solid or liquid materials are significantly broader than gas absorption features but are still covered in the broad spectral range of SCLAS. Thus applying the measured initial intensity distribution in the evaluation would introduce a low frequency distortion in the signal. Furthermore the short time scales of the laser pulses make an accurate tuning of the light path lengths of the reference beam path to the measurement beam path necessary. Otherwise the two measurements would be shifted on the time axis. To avoid these disadvantages the procedure presented here does not rely on a reference measurement, but uses a fitted background model for evaluation.

Current challenges in the SCLAS data analysis are the high absorption line count that needs to be evaluated, the determination and application of the instrument function and the unknown initial intensity distribution prior to absorption. The high number of absorption lines of a species that is covered in a single measurement on the one hand improves measurement precision. On the other hand it makes the evaluation using a model fitting approach computationally very expensive. Regarding the achieved spectral resolutions^7,11,12^ in the order of 0.1 cm^−1^ the instrument function of a SCLAS spectrometer can not be neglected. An important influence on the instrument function is the photo diode whose influence can become visible as so called diode ringing^11^. Blume *et al*. neglect the instrument function and evaluate only the line areas. Therefore this approach does not allow for the evaluation of pressure values. Werblinski *et al*. determine the instrument function of the photo diode by a separate measurement, but the influence of the initial SCL pulse length and profile are neglected. To overcome these disadvantages a comprehensive approach has been developed for the *in-situ* determination of an instrument function covering all effects given by the SCL source, the photo diode and the data acquisition. Furthermore many of the current evaluation procedures evaluate the data in the absorbance domain and not in the intensity domain. Hence a discussion of the advantages of the evaluation in the intensity domain is given. These advantages mainly become visible in measurements with strong absorption, which are often unavoidable due to boundary conditions of the experiment. Regarding the unknown initial intensity distribution a simultaneous fitting approach^13^ is applied, while previous evaluation procedures used estimation algorithms for the background^11,14^. These components are combined to a comprehensive SCLAS data analysis method, that enables the evaluation of mole fraction, temperature and pressure from SCLAS measurements even for partial total absorption. To estimate the potential of SCLAS for measuring different quantities under various conditions a detailed uncertainty estimation regarding noise, uncertainty in wavenumber axis and uncertainty in the instrument function is given.

## Methods

For the processing of broadband absorption spectroscopic measurements different methods have been proposed. These methods can be distinguished regarding the amount of physical knowledge that is utilized in the data analysis. Statistical methods like partial least squares (PLS) do not require any knowledge of the physical system but rather calibrate a purely statistical model using several calibration measurements^15^. Hybrid methods like indirect hard modeling (IHM) utilize physical knowledge, like line shape functions, to reduce the measurement data and calibrate a more robust statistical model^16^. An approach that does not depend on calibration data is the fitting of a comprehensive physical model. This model can be derived from a physical model and certain fixed parameters (e.g. line data). This approach has already been applied to SCLAS data^8,11,14^. The preference for the physical model approach is easily explained regarding the availability of line databases like HITRAN^17^ and the possibility to avoid calibration measurements. Therefore the method explained in this article will also follow this approach.

In the following sections a model for the measured voltage signal is developed, that incorporates the actual absorption effects as well as the behaviour of DCM, photo diode and oscilloscope.

### Absorbance calculation

The first part of this physical model is a model for the absorbance depending on the temperature *T*, the pressure *p* and the absorber mole fraction *q*. The line data that is needed for such a simulation can be taken from databases like HITRAN2016^17^, which is used in this work. According to Beer-Lambert’s law the absorbance of several absorption transitions can be described as

1 $$A(\nu ,T,p,q)=-\,\mathrm{ln}\,\frac{{I}_{\nu }(\nu ,T,p,q)}{{I}_{\nu ,0}(\nu )}=qNL\,\sum _{i}{S}_{i}(T){\rm{\Phi }}(\nu -{\nu }_{0,i},{\rm{\Delta }}{\nu }_{{\rm{G}},i},{\rm{\Delta }}{\nu }_{{\rm{L}},i}).$$

In equation (1), *I*_*ν*_ is the transmitted spectral intensity at wavenumber *ν*, *I*_*ν*,0_ is the initial spectral intensity, *q* is the absorber mole fraction, *L* is the absorption path length, *S*_*i*_(*T*) is the line strength of the line *i*, Φ(*ν*, *p*, *T*, *q*) is the line profile function and *ν*_0,*i*_ is the center frequency of transition *i* (already regarding pressure shifts). *N* describes the number density in the measured gas sample, which in the case of ideal gas can be calculated as *N* = *qp*/(*k*_b_*T*) with the Boltzmann constant *k*_b_. For many technical systems the line profile function Φ(*ν*, Δ*ν*_G,*i*_, Δ*ν*_L,*i*_) is sufficiently described by a Voigt profile, which is a convolution of a Gauss profile with a full-width-at-half-maximum (FWHM) Δ*ν*_G,*i*_ and a Lorentzian with a FWHM Δ*ν*_L,*i*_. These profile widths are calculated using line data supplied by HITRAN and therefore are limited to self- and air-broadening.

Using HITRAN reference line strengths *S*_*i*_(*T*_0_), the line strength at temperature T can be calculated as

2 $${S}_{i}(T)={S}_{i}({T}_{0})\frac{Z({T}_{0})}{Z(T)}\frac{\exp (-{E}_{i}^{^{\prime\prime} }/({k}_{{\rm{b}}}T))}{\exp (-{E}_{i}^{^{\prime\prime} }/({k}_{{\rm{b}}}{T}_{0}))}\cdot \frac{1-\exp (-hc{\nu }_{0,i}/({k}_{{\rm{b}}}T))}{1-\exp (-hc{\nu }_{0,i}/({k}_{{\rm{b}}}{T}_{0}))}.$$

In equation (2), *Z*(*T*) is the total partition sum at temperature *T*, *E*″_*i*_ is the energy of the lower state of transition *i* and *h* is Planck’s constant.

The calculation of absorbance spectra for the evaluation of SCLAS data can be computationally very expensive, because the number of lines to be simulated can easily reach several thousands (depending on the analysed species and spectral coverage). The calculation of the Voigt profile constitutes the largest part of the calculations during the absorbance simulation. Therefore the Voigt approximation approach of McLean *et al*.^18^ was chosen for its reduced computational effort.

### Background intensity

The initial or background intensity *I*_0_ of the SCLAS measurement can either be measured simultaneously^8^ or be estimated from the actual measurement signal. In the SCLAS spectrometer regarded in this work, the incident background intensity *I*_0_ before absorption is not measured separately or simultaneously. For modeling this unknown background intensity, different approaches are possible. The first approach is to estimate the background using only the measured *I* with absorption lines^11^. This approach relies either on a certain shape of the absorption lines or on a difference in the frequency content of absorption lines and the background. According to an approach originally developed by Kranendonk *et al*. the derivative of the intensity $\frac{{\rm{d}}I}{{\rm{d}}\nu }$ is evaluated instead of the intensity itself. This way the low frequency content of the background is damped while the higher frequency content of the absorption lines is emphasized in the fitting process. This approach has already been applied to SCLAS measurements in combination with a measured *I*_0_^8^. While these two approaches either estimate the background or limit the influence of the background on the fitting result, it is also possible to create a background model that is fitted to the measurement data simultaneously^13^. This approach promises good accuracy and is already used commonly in TDLAS data analysis. In the context of SCLAS the fitting process grows in complexity due to the additional background variables that need to be optimized. Nonetheless the simultaneous fitting approach was chosen for the implementation in the current work as it promises the best results. Therefore the raw model of the intensity is described by

3 $${I}_{{\rm{R}}}(\nu ,T,p,q,{\beta }_{0},\ldots {\beta }_{m})={I}_{0}(\nu ,{\beta }_{0},\ldots {\beta }_{m})\,\exp \,(-A(\nu ,T,p,q)).$$

Here *β*_0_, …, *β*_*m*_ are the background parameters, that define the shape of the background. Polynomials are often chosen as parametric background functions^13^ but due to the strong fluctuations in the SCL intensity distribution the polynomial approach is not expected to describe the profile sufficiently accurate. Therefore a cubic spline function with equidistant support points is chosen to describe the background. The number of support points of the spline has to be adapted to the problem and the regarded spectral window. It has to be high enough to describe the background sufficiently, but if the number of support points is chosen too high the background tends to partly approximate the absorption lines. This effect can influence the fitting results and has to be avoided. As of now the maximum allowable number of support points has to be estimated according to the maximum expected absorption line FWHM Δ*ν*_max_. Under the assumption that collisional broadening is the dominant broadening mechanism the maximum line widths are reached at maximum expected pressure and lowest expected temperature. For these parameters the Lorentz FWHM of all absorption lines in the regarded range are calculated. As collisional broadening is also dependent on the collision partner, these widths have to be calculated twice: once for absorber concentrations approaching zero (e.g. *q* = 0.001) and once for a pure absorber (*q* = 1). The largest Lorentz FWHM of both cases is used as Δ*ν*_max_. As a rule of thumb the distance between the equidistant spline support point should not be chosen smaller than Δ*ν*_max_.

### Instrument function

The instrument function of the SCLAS spectrometer is influenced by different parts of the system: the finite length and temporal pulse profile of the undispersed SCL pulse, the dynamic response of the high bandwidth photo diode and the finite sampling rate and pulse response of the oscilloscope. The sampling rate is known and the pulse response function of the high bandwidth photo diode is either supplied by the manufacturer or can be measured using pulsed lasers^14,19^. Nonetheless the SCL pulse profile is still unknown and the transfer of the manufacturer supplied photo diode response function to a different measurement system with even minimal deviations in the electrical characteristics is critical. Therefore we present an approach for the determination of the instrument function directly from a measurement with known absorber mole fraction, temperature and pressure. The three already mentioned influences on the instrument function will be discussed separately before finally describing the combined method. Note that in the following discussion transmission loses other than the absorption in the measurement volume will be neglected because they do not influence the spectral resolution or instrument function in the absorbance signal.

#### Influence of the finite SCL pulse

The initial SCL pulse spectrally typically covers wavenumber regions that are not needed for the actual measurement. Therefore the pulse is bandpass filtered for the spectral region of interest in order to maximize the achievable repetition rate. This filtering does not influence the instrument function as it only limits the covered spectral region. The resultant SCL pulse can be described by a time dependent spectral intensity distribution *I*_*ν*,SCL_ (*t*, *ν*). Due to dispersion and other effects during super continuum generation a significant wavelength dependent delay can be introduced. In other words, the peak wavelength is dependent on time during the pulse. To simplify the modelling of the SCL pulse this wavelength dependent delay is incorporated into the model of the DCM discussed below. Thus the model for *I*_*ν*,SCL_ (*t*, *ν*) discussed here neglects this time dependency of the spectral content of the pulse.

Therefore the qualitative spectral intensity distribution of the pulse is assumed to stay constant over the whole pulse duration while only the intensity is dependent on time. Put differently the normalized spectral distribution ${\hat{I}}_{\nu ,{\rm{SCL}}}(\nu )$ of the pulse is assumed to be independent of time:

4 $${\hat{I}}_{\nu ,{\rm{SCL}}}(\nu )=\frac{{I}_{\nu ,{\rm{SCL}}}(t,\nu )}{{I}_{{\rm{SCL}}}(t)}={\rm{const}},\,{\rm{with}}\,{I}_{{\rm{SCL}}}(t)={\int }_{0}^{\infty }{I}_{\nu ,{\rm{SCL}}}(t,\nu ){\rm{d}}\nu .$$

This assumption allows the spectral intensity distribution *I*_*ν*,SCL_ (*t*, *ν*) to be described by

5 $${I}_{\nu ,{\rm{SCL}}}(t,\nu )={I}_{{\rm{SCL}}}(t){\hat{I}}_{\nu ,{\rm{SCL}}}(\nu ).$$

Note that this model of the initial SCL pulse is still a rough approximation of the SCL pulse, but allows for a simple derivation of the instrument function and gives good results in practical application as will be shown in the experimental results.

**Figure 2:**
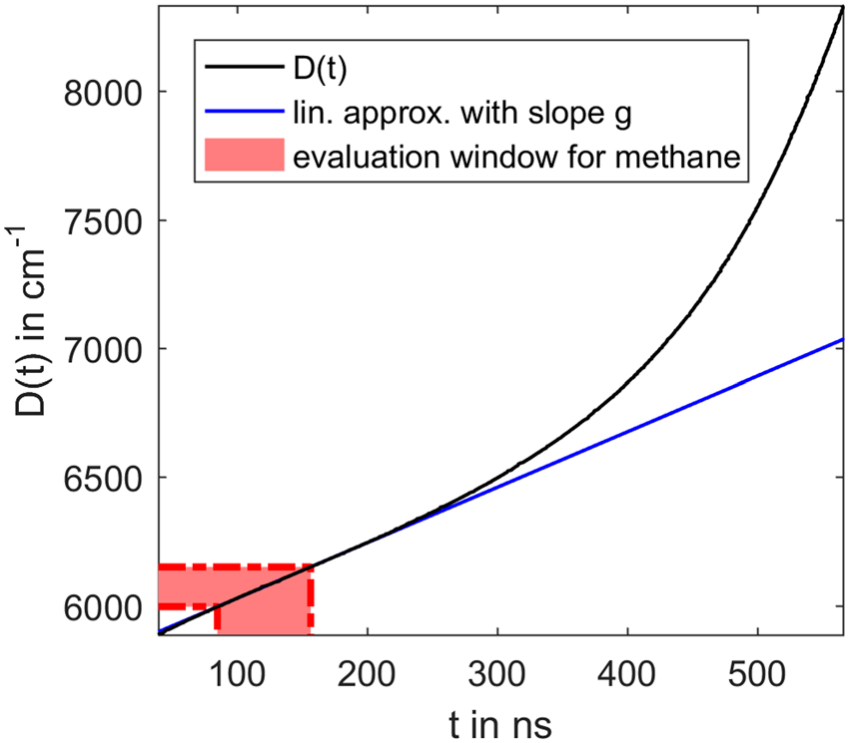
The time-wavenumber relation. The function *D*(*t*) assigns the corresponding wavenumber to the time points in the measurement.

By passing through the DCM the spectral components of the pulse are delayed according to their wavenumber. Hence a function *D* : *t* → *ν* can be defined, that assigns a wavenumber to every time step in the measurement. An example for this function *D*(*t*) is given in Fig. 2. As already mentioned this function also incorporates wavelength dependent delays introduced during the super continuum generation. A estimate of this function can be accquired using methods described by Blume *et al*.^6^. But as *D*(*t*) needs to be known accurately, a method using the absorption lines as a reference is applied^11^. Using the DCM dependent function *D*(*t*) the time dependent spectral intensity distribution after the DCM is described by

6 $${I}_{\nu ,{\rm{DCM}}}(t,\nu )={I}_{\nu ,{\rm{SCL}}}(t-{D}^{-1}(\nu ),\nu )={I}_{{\rm{SCL}}}(t-{D}^{-1}(\nu )){\hat{I}}_{\nu ,{\rm{SCL}}}(\nu )$$

After the DCM the dispersed pulse is passed through the measurement volume and partially absorbed:

7 $${I}_{\nu ,{\rm{ABS}}}(t,\nu )={I}_{{\rm{SCL}}}(t-{D}^{-1}(\nu )){\hat{I}}_{\nu ,{\rm{SCL}}}(\nu )\,{{\rm{e}}}^{-A(\nu )}.$$

#### Influence of the photo diode

The dispersed pulse, modulated with the absorption features (equation (7)) is then detected by the photo diode. Note that in the current context *photo diode* refers to the whole detection system including the measurement amplifier as it significantly influences the response of the photo diode. Furthermore only the dynamic response of the photo diode is regarded in the following section.

The wavelength dependent sensitivity of the photo diode will be neglected at this point as it does not have any effect on the instrument function such that only the time dependent pulse response of the photo diode is regarded. Thus it is reasonable to only consider the time dependent intensity *I*_ABS_(*t*) at the photo diode instead of the spectral intensity distribution. The intensity *I*_ABS_(*t*) is defined by the integral

8 $${I}_{{\rm{ABS}}}(t)={\int }_{0}^{\infty }{I}_{{\rm{SCL}}}(t-{D}^{-1}(\nu )){\hat{I}}_{\nu ,{\rm{SCL}}}(\nu )\,{{\rm{e}}}^{-A(\nu )}\,{\rm{d}}\nu .$$

The normalized spectral intensity distribution ${\hat{I}}_{\nu ,{\rm{SCL}}}(\nu )$ is always limited to a certain wavenumber range defined by the capabilities of the SCL itself. Outside of this wavenumber range ${\hat{I}}_{\nu ,{\rm{SCL}}}(\nu )$ is zero. Therefore an expansion of the lower integration limit in equation (8) to −∞ will not change the value of the integral. Hence equation (8) can be rewritten to

9 $${I}_{{\rm{ABS}}}(t)={\int }_{-\infty }^{\infty }{I}_{{\rm{SCL}}}(t-{D}^{-1}(\nu )){\hat{I}}_{\nu ,{\rm{SCL}}}(\nu ){{\rm{e}}}^{-A(\nu )}{\rm{d}}\nu \mathrm{.}$$

The relation between time delay and wavelength is given by *D*(*t*). Thus Equation (9) can be transferred to the time domain by a substitution in the integral:

10 $$\begin{array}{rcl}{I}_{{\rm{ABS}}}(t) & = & {\int }_{-\infty }^{\infty }{I}_{{\rm{SCL}}}(t-{D}^{-1}(\nu )){\hat{I}}_{\nu ,{\rm{SCL}}}(\nu )\,{{\rm{e}}}^{-A(\nu )}{\rm{d}}\nu \\  & = & {\int }_{-\infty }^{\infty }{I}_{{\rm{SCL}}}(t-\tau ){\hat{I}}_{\nu ,{\rm{SCL}}}(D(\tau ))\,{{\rm{e}}}^{-A(D(\tau ))}\frac{{\rm{d}}D(\tau )}{{\rm{d}}\tau }{\rm{d}}\tau .\end{array}$$

As *I*_SCL_(*t*) describes a short pulse in the vicinity of *t* = 0, it is valid to assume that *I*_SCL_(*t*) is only non-zero in a short time interval 0 ≤ *t* ≤ Δ*t*_SCL_. Thus for a constant time *t* the integral in equation (8) is only dependent on a interval in the time shift *τ* of length Δ*t*_SCL_. The pulse length Δ*t*_SCL_ is in the order of 100 ps and thus too short for any significant change in $\frac{{\rm{d}}D(\tau )}{{\rm{d}}\tau }$ (see Fig. 2). Therefore for every *t* in equation (10) the derivative $\frac{{\rm{d}}D(\tau )}{{\rm{d}}\tau }$ in the integral can be assumed to be a constant $\frac{{\rm{d}}D(\tau )}{{\rm{d}}\tau }$. Equation (8) can thus be rewritten to

11 $${I}_{{\rm{ABS}}}(t)=\frac{{\rm{d}}D(t)}{{\rm{d}}t}{\int }_{-\infty }^{\infty }{I}_{{\rm{SCL}}}(t-\tau ){\hat{I}}_{\nu ,{\rm{SCL}}}(D(\tau ))\,{{\rm{e}}}^{-A(D(\tau ))}{\rm{d}}\tau \mathrm{.}$$

Equation (11) describes a convolution in the time domain.

With these integration limits the integral describes a convolution. Therefore for all *t* ≥ 0 equation (11) can be written

12 $$\begin{array}{rcl}{I}_{{\rm{ABS}}}(t) & = & \frac{{\rm{d}}D(t)}{{\rm{d}}t}{\int }_{-\infty }^{\infty }{I}_{{\rm{SCL}}}(t-\tau ){\hat{I}}_{\nu ,{\rm{SCL}}}(D(\tau ))\,{{\rm{e}}}^{-A(D(\tau ))}{\rm{d}}\tau \\  & = & {I}_{{\rm{SCL}}}(t)\ast [\frac{{\rm{d}}D(t)}{{\rm{d}}t}{\hat{I}}_{\nu ,{\rm{SCL}}}(D(t))\,{{\rm{e}}}^{-A(D(t))}],\end{array}$$

with * being a convolution. The factor $\frac{{\rm{d}}D(t)}{{\rm{d}}t}$ in equation (12) describes the trade-off between spectral resolution and signal intensity. A higher spectral resolution requires the SCL pulse to be further dispersed in time. This is equivalent to a lower derivative $\frac{{\rm{d}}D(t)}{{\rm{d}}t}$ as in an equal time less wavenumbers should be scanned. With a lower $\frac{{\rm{d}}D(t)}{{\rm{d}}t}$ the intensity *I*_ABS_(*t*) at the photo diode decreases. This behaviour is intuitively explained as the SCL pulse only consists of a limited amount of energy. Thus an elongation of the pulse in time results in a smaller intensity.

Furthermore the factor $\frac{{\rm{d}}D(t)}{{\rm{d}}t}$ can be assumed to be constant during a typical signal window for evaluation (e.g. for methane 5995 cm^−1^ to 6145 cm^−1^). Thus the derivative $\frac{{\rm{d}}D(t)}{{\rm{d}}t}$ can be replaced by a constant mean value

13 $$g=\frac{1}{{t}_{2}-{t}_{1}}{\int }_{{t}_{1}}^{{t}_{2}}\frac{{\rm{d}}D(\tau )}{{\rm{d}}\tau }\,{\rm{d}}\tau =const.$$

Here *t*_1_ and *t*_2_ describe the regarded measurement window. This approximation is illustrated in Fig. 2. Note that even if the change in $\frac{{\rm{d}}D(t)}{{\rm{d}}t}$ was significant, the only effect would be a relatively slow variation of the intensity signal that does not affect the instrument function or the evaluation. In fact this variation could not be distinguished from variations in the initial intensity distribution or transmission properties of e.g. optical windows in the experimental setup. Therefore the final description of the intensity at the photo diode is

14 $${I}_{{\rm{ABS}}}(t)={I}_{{\rm{SCL}}}(t)\ast [g{\hat{I}}_{\nu ,{\rm{SCL}}}(D(t))\,{{\rm{e}}}^{-A(D(t))}]={I}_{{\rm{SCL}}}(t)\ast [{I}_{0}(D(t))\,{{\rm{e}}}^{-A(D(t))}\mathrm{].}$$

The photo diode itself is modeled as a linear time invariant system and is therefore fully described by its pulse response *h*_PD_(*t*)^20^. The resulting voltage at the photo diode is given as the convolution

15 $${U}_{{\rm{PD}}}(t)={h}_{{\rm{PD}}}(t)\ast {I}_{{\rm{ABS}}}(t)={h}_{{\rm{PD}}}(t)\ast {I}_{{\rm{SCL}}}(t)\ast [{I}_{0}(t)\,{{\rm{e}}}^{-A(D(t))}\mathrm{].}$$

#### Influence of the data acquisition

Similar to the photo diode the finite sampling rate and pulse response of the oscilloscope is modelled as another pulse response *h*_DAQ_(*t*):

16 $$\begin{array}{rcl}{U}_{{\rm{DAQ}}}(t) & = & {h}_{{\rm{DAQ}}}(t)\ast {U}_{{\rm{PD}}}(t)={h}_{{\rm{DAQ}}}(t)\ast {h}_{{\rm{PD}}}(t)\ast {I}_{{\rm{SCL}}}(t)\ast [{I}_{0}(t)\,{{\rm{e}}}^{-A(D(t))}]\\  & = & {h}_{{\rm{SCLAS}}}(t)\ast [{I}_{0}(t)\,{{\rm{e}}}^{-A(D(t))}],\end{array}$$

where *h*_SCLAS_(*t*) is the pulse response of the whole spectrometer.

Due to the stochastic process involved in the creation of a SCL pulse, the spectral intensity distribution of a single pulse and therefore *I*_0_(*ν*) is highly unsteady. Thus several pulses have to be averaged in accordance with the trigger of the pump pulse:

17 $$\begin{array}{rcl}{\bar{U}}_{{\rm{DAQ}}}(t) & = & \frac{1}{n}\,\sum _{i=1}^{n}{h}_{{\rm{SCLAS}}}(t)\ast [{I}_{\mathrm{0,}i}(t)\,{{\rm{e}}}^{-A(D(t))}]={h}_{{\rm{SCLAS}}}(t)\ast \frac{1}{n}\sum _{i=1}^{n}{I}_{\mathrm{0,}i}(t)\,{{\rm{e}}}^{-A(D(t))}\\  & = & {h}_{{\rm{SCLAS}}}(t)\ast {\bar{I}}_{0}(t){{\rm{e}}}^{-A(D(t))}.\end{array}$$

In equation (17), the measured pulses $[{I}_{\mathrm{0,}i}(t){{\rm{e}}}^{-A(D(t))}]$ are shifted such that *t* = 0 always refers to the trigger of the corresponding pulse. Furthermore equation (17) assumes that the absorbance *A*(*D*(*t*)) is constant and thus that the gas state does not change during the measurement interval. The averaging results in a less noisy averaged measurement signal ${\bar{U}}_{{\rm{DAQ}}}(t)$ due to the less noisy, averaged initial intensity distribution ${\bar{I}}_{0}(t)$. The Comparison of equation (17) to equation (16) shows that the averaging has no influence on the instrument function. Thus the averaged signal and the signal of a single pulse can be treated similarly in the evaluation.

#### Determination of the instrument function

According to equation (16) all instrumental influences on the measurement signal can be described by a convolution with a certain pulse response *h*_SCLAS_ in the time domain. Due to the discrete sampling of the oscilloscope, measurements are only available at the time points *t*_*l*_ = *l*Δ*t*_sampling_ with $l\in {\mathbb{Z}}$. Thus the continuous convolution is approximated as a discrete one:

18 $${U}_{{\rm{DAQ}}}[l]={h}_{{\rm{SCLAS}}}[l]\ast [{I}_{0}[l]\,{{\rm{e}}}^{-A(D[l])}\mathrm{].}$$

To determine *h*_SCLAS_ [*l*] from measurements, *U*_DAQ_[*l*] and *I*_0_[*l*] e^−*A*(*D*[*l*])^ have to be known. *U*_DAQ_[*l*] is easily attained as it is a SCLAS measurement, whereas *I*_0_[*l*] e^−*A*(*D*[*l*])^ can not be measured directly. Therefore *I*_0_[*l*] e^−*A*(*D*[*l*])^ needs to be simulated according to theory. Thus SCLAS measurements with known temperature, pressure and mole fraction are used, which allow the simulation of *A*(*ν*) from HITRAN data. The background intensity distribution model *I*_0_[*l*] (a spline function) is then fitted to the measurement using an estimated instrument function. The result is a discrete deconvolution problem with the measured *U*_DAQ_[*l*] and the simulated *I*_th_ = *I*_0_[*l*] e^−*A*(*D*[*l*])^. The discrete convolution can be described as a linear equation system

19 $$\begin{array}{ccc}(\begin{array}{c}\vdots \\ {U}_{{\rm{D}}{\rm{A}}{\rm{Q}}}[i]\\ {U}_{{\rm{D}}{\rm{A}}{\rm{Q}}}[i+1]\\ \vdots \end{array}) & = & (\begin{array}{ccccc}\ddots  & \vdots  & \vdots  & \vdots  & \\ {I}_{{\rm{t}}{\rm{h}}}[i-\mu ] & \ldots  & {I}_{{\rm{t}}{\rm{h}}}[i] & \ldots  & {I}_{{\rm{t}}{\rm{h}}}[i+\mu ]\\ {I}_{{\rm{t}}{\rm{h}}}[i-\mu +1] & \ldots  & {I}_{{\rm{t}}{\rm{h}}}[i+1] & \ldots  & {I}_{{\rm{t}}{\rm{h}}}[i+\mu +1]\\  & \vdots  & \vdots  & \vdots  & \ddots \end{array})\cdot (\begin{array}{c}{h}_{{\rm{S}}{\rm{C}}{\rm{L}}{\rm{A}}{\rm{S}}}[-\mu ]\\ \vdots \\ {h}_{{\rm{S}}{\rm{C}}{\rm{L}}{\rm{A}}{\rm{S}}}[0]\\ {h}_{{\rm{S}}{\rm{C}}{\rm{L}}{\rm{A}}{\rm{S}}}[1]\\ \vdots \\ {h}_{{\rm{S}}{\rm{C}}{\rm{L}}{\rm{A}}{\rm{S}}}[\mu ]\end{array})\\  & = & {\bf{A}}\cdot {{\bf{h}}}_{{\rm{S}}{\rm{C}}{\rm{L}}{\rm{A}}{\rm{S}}}.\end{array}$$

It is valid to assume that the SCLAS pulse response function is narrow compared to the actual measurement duration. Thus non-zero elements in the vector **h**_SCLAS_ are limited to a small range −*μ* ≤ *l* ≤ *μ* with $\mu \in {\mathbb{Z}}$ symmetrical around *t* = *l* = 0. The elements outside of this range are neglected. With $n\gg (2\mu +1)$ measurement samples the *n* × (2*μ* + 1) matrix **A** describes an overdetermined linear equation system for which the least square solution **h**_SCLAS_ can be found. This yields a valid estimate for the instrument function that can be used for different measurements with the same setup. The best value for *μ* has to be determined empirically. Too low values will cut away parts of the pulse response function and therefore distort the profile. Too high values increase the number of variables and thus increase the instability of the equation system and the influence of noise on the result.

### Cost function and fitting

The cost function is chosen to compare modelled voltage *U*_OSC_ and measured voltage ${\hat{U}}_{{\rm{OSC}}}$ at all measurement points *i*:

20 $$Z(T,p,q,{\beta }_{0},\ldots ,{\beta }_{n})=\sum _{i}{z}_{i}^{2}=\sum _{i}\frac{{({U}_{{\rm{DAQ}}}({t}_{i},T,p,q,{\beta }_{0},\ldots ,{\beta }_{n})-{\hat{U}}_{{\rm{DAQ}}}({t}_{i}))}^{2}}{{\sigma }_{i}^{2}}$$

The measurement uncertainties *σ*_*i*_ at the time points *t*_*i*_ are assumed to be equal *σ*_*I*_ = *σ*_*i*_. As the standard deviation *σ*_*I*_ can not be estimated beforehand, it is set to *σ*_*I*_ = 1, because the result of the fitting is not dependent on a constant factor in the cost function. Whereas the commonly used cost function compares absorbances^13^, equation (20) directly compares the measured voltage values to simulated ones. The advantage of this domain for comparison emerges when strong absorbance peaks are evaluated. The uncertainty *σ*_A_ of an absorbance value due to an intensity (or equivalent voltage) uncertainty *σ*_*I*_ is given by equation (1) and Gaussian error propagation as

21 $${\sigma }_{{\rm{A}}}^{2}=\frac{1}{{I}^{2}}{\sigma }_{I}^{2}.$$

Hence the absorbance uncertainty increases inversely to the measured intensity (or voltage). Thus strong absorption lines with a small measured intensity lead to big uncertainties in the absorbance, that are not considered in the cost function as they are not known beforehand. Therefore the direct evaluation of the measured voltage promises better evaluation results in measurements with strong absorption features.

The cost function is minimized using the non-linear least-square optimizer function lsqnonlin in MATLAB. To increase the numerical efficiency of the optimization the Jacobian of the signal model *U*_DAQ_ is calculated directly and supplied to the optimization algorithm.

**Figure 3:**
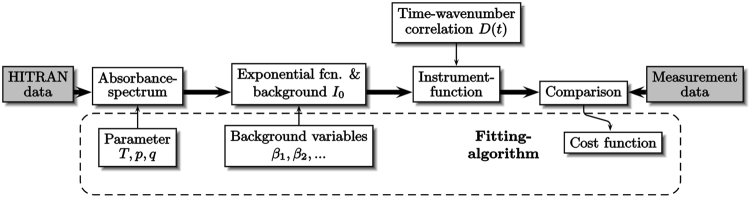
Flow diagram of the evaluation and fitting procedure. A signal model with temperature, pressure, mole fraction as well as several background variables as parameters is iteratively fitted to a measurement signal.

The optimization process yields the optimum values *T*′, *p*′, *q*′ and *β*_*j*_ for the parameters *T*, *p*, *q* and *β*_*j*_. These values minimize the cost function and therefore represent the evaluation result of the measurements in temperature, pressure and mole fraction. This evaluation procedure is summarized in Fig. 3 including all model components with their respective parameters.

### Fit uncertainty estimation

To evaluate the capabilities of the SCLAS measurement and analysis method, the resulting uncertainty of the model fitting approach is calculated. Uncertainties of the HITRAN parameters are not an essential part of the SCLAS measurement and analysis method described here and can be replaced with higher quality line data. Therefore the uncertainties of the HITRAN parameters are neglected in the following uncertainty estimation to allow for an unbiased estimation of the potential of SCLAS. Put differently, the uncertainty estimation presented in this paper sets a maximum limit for the achievable precision in the regarded SCLAS setup by assuming perfect line data. The practically achievable precision and accuracy of course still depend on the quality of the line data.

The uncertainty of the SCLAS measurement and analysis can be divided into the uncertainty induced by measurement noise, by the determination of the wavenumber axis and by the instrument function. These factors will be discussed separately and are combined in the end.

To determine the influence of the noise of the intensity measurement on the fitted parameters *p*′, *T*′ and *q*′, the variance of the measurement noise is estimated. With *k* sampled points and *m* fitted variables *a*_*j*_ (*p*, *T*, *q* and *β*_0_, …, *β*_*n*_) the standard deviation of the signal noise is estimated to be:

22 $${\sigma }_{I}={[\frac{1}{k-m}\sum _{i}{r}_{i}^{2}]}^{\frac{1}{2}},$$

where *r*_*i*_ is the final fit residuum

23 $${r}_{i}={\hat{U}}_{{\rm{DAQ}}}({t}_{i})-{U}_{{\rm{DAQ}}}({t}_{i},T,p,q,{\beta }_{0},\ldots ,{\beta }_{n}).$$

Equation (22) considers the reduction of the degrees of freedom of the residuum by the number of fitted parameters *m*. The determination of fitting parameter uncertainties from measurement data uncertainties is well known for linear fits. But the nonlinear model demands the consideration of inter-parameter dependencies. According to Burell^21^ the uncertainties ${\sigma }_{{a}_{j},noise}$ of the determined fit parameters *a*_*j*_ for this non-linear model can be calculated as

24 $${\sigma }_{{a}_{j},noise}^{2}=\sum _{i=1}^{k}\,{[\sum _{l=1}^{m}\frac{1}{\sigma }{\varepsilon }_{jl}\frac{\partial }{\partial {a}_{l}}{U}_{{\rm{DAQ}}}({t}_{i},{\bf{a}})]}^{2}.$$

where the matrix *ε*_*jl*_ is the inverse of the matrix *α*_*jl*_:

25 $${({\varepsilon }_{jl})}^{-1}={\alpha }_{jl}=\sum _{i=1}^{k}\,\frac{1}{{\sigma }^{2}}\,[(\frac{\partial }{\partial {a}_{j}}{U}_{{\rm{DAQ}}}({t}_{i},{\bf{a}}))\cdot (\frac{\partial }{\partial {a}_{l}}{U}_{{\rm{DAQ}}}({t}_{i},{\bf{a}}))-{r}_{i}\frac{{\partial }^{2}}{\partial {a}_{j}\partial {a}_{l}}{U}_{{\rm{DAQ}}}({t}_{i},{\bf{a}})].$$

The model derivatives of first and second order are estimated using finite differences.

The experimentally determined time-wavelength-relation *D*(*t*) is also subject to measurement uncertainty. This uncertainty is similar to an uncertainty in the measurement times *t*_*i*_. Solutions to this uncertainty problem for nonlinear models are discussed by Cecchi^22^ under the assumption of uncorrelated uncertainties. But the assumption of uncorrelated errors in the measurement time *t*_*i*_ is not valid for this case since the deviations are correlated by the function *D*(*t*). Hence the methods applied by Burrell and Cecchi need to be adopted to account for this correlation. First an assumption concerning the correlation has to be made. One systematic source of such a correlated error could be introduced by the determination of *D*(*t*). But the results for *D*(*t*) lead to no visible deviations between measured absorption lines and HITRAN line positions. Even if a small deviation was present, the method used for the determination of the instrument function is able to compensate small and constant deviations in the wavenumber axis. A possible source for a stochastic error in the wavenumber axis is given by jittering of the trigger signal for the data acquisition. A deviation in the trigger time would lead to a constant offset in *D*(*t*) as all measurement times *t*_*i*_ would be shifted equally. This error source is assumed to be dominant. Therefore the dispersion function is decomposed to

26 $$D(t)={D}_{0}+D^{\prime} (t).$$

Only the constant offset *D*_0_ is assumed to have an uncertainty, which leads to a constant and therefore directly correlated offset in the wavenumber axis. With this definition *D*_0_ becomes an additional parameter of the simulated signal *U*_DAQ_(*t*_*i*_, **a**, *D*_0_).

After a laser warm up phase of two hours for the NKT SuperK EXTREME EXW-12 no trigger jitter could be measured within the resolution of the oscilloscope (LeCroy SDA 820Zi-A oscilloscope, 40 GSps, 20 GHz bandwidth). Thus the uncertainty in *D*_0_ is limited by the time step size respectively the sampling frequency. The function *D*(*t*) can be approximated as linear in the measurement window and the values of *D*_0_ are assumed to be uniformly distributed in an interval of the width *g*Δ*t*_sampling_ with *g* defined according to equation (13). Hence the standard deviation of *D*_0_ is estimated to be^23^

27 $${\sigma }_{{D}_{0}}=\frac{1}{\sqrt{12}}g{\rm{\Delta }}{t}_{{\rm{sampling}}}.$$

To determine the influence of the uncertainty *σ*_*D*0_ on the resulting optimum parameters the implicit description of the fitting optimum^21,22^

28 $$\frac{\partial Z}{\partial {a}_{j}}=0,\,{\rm{with}}\,j=1,2,\ldots ,m$$

is considered. This implicit description is differentiated with respect to *D*_0_ and yields (see^21,22^)

29 $$\frac{{\partial }^{2}Z}{\partial {a}_{j}\partial {D}_{0}}+\sum _{l=1}^{m}\frac{{\partial }^{2}Z}{\partial {a}_{l}\partial {a}_{j}}\frac{\partial {a}_{l}}{\partial {D}_{0}}=\mathrm{0,}\,j=\mathrm{1,}\,\mathrm{2,}\,\mathrm{...,}\,m\mathrm{.}$$

With equation (20) and *σ*_*i*_ = *σ*, equation (29) becomes

30 $$\sum _{l\mathrm{=1}}^{m}(\frac{\partial {a}_{l}}{\partial {D}_{0}})\sum _{i=1}^{k}[\frac{\partial {U}_{{\rm{DAQ}}}({t}_{i})}{\partial {a}_{l}}\frac{\partial {U}_{{\rm{DAQ}}}({t}_{i})}{\partial {a}_{j}}-{r}_{i}\frac{{\partial }^{2}{U}_{{\rm{DAQ}}}({t}_{i})}{\partial {a}_{l}\partial {a}_{j}}]=-\,\sum _{i=1}^{k}[\frac{\partial {U}_{{\rm{DAQ}}}({t}_{i})}{\partial {D}_{0}}\frac{\partial {U}_{{\rm{DAQ}}}({t}_{i})}{\partial {a}_{j}}-{r}_{i}\frac{{\partial }^{2}{U}_{{\rm{DAQ}}}({t}_{i})}{\partial {a}_{j}\partial {D}_{0}}]$$

with *j* = 1, 2, ..., *m*. As the derivatives of *U*_DAQ_ can again be estimated using finite differences, equation (30) describes a linear equation system for the derivatives ∂*a*_*j*_/∂*D*_0_. With these derivatives the uncertainty in *a*_*j*_ induced by the uncertainty in *D*_0_ can be calculated by classical error propagation as

31 $${\sigma }_{{a}_{j},D}^{2}={(\frac{\partial {a}_{j}}{\partial {D}_{0}})}^{2}{\sigma }_{{D}_{0}}^{2}\mathrm{.}$$

Additionally the uncertainty due to the instrument function has to be considered. Thus the uncertainty in the instrument function itself has to be calculated first. Because the determination of the instrument function itself is a linear least square fit, the uncertainties *σ*_if,*l*_ can be determined with the classical uncertainty estimation for least squares^21^. These uncertainties are propagated to the actual model by the discrete convolution in equation (18). The resulting uncertainty *σ*_U,if,*i*_ of the model *U*_DAQ_ at the time *t*_*i*_ regarding all uncertainties *σ*_if,*l*_ is

32 $${\sigma }_{{\rm{U}},{\rm{if}},i}^{2}=\sum _{l=-\varepsilon }^{\varepsilon }{({U}_{{\rm{DAQ}}}({t}_{i-l}){\sigma }_{{\rm{if}},{\rm{l}}})}^{2}\mathrm{.}$$

Similarly to the application of Burrell’s approach in the wavenumber uncertainty assessment, the differentiation of the implicit equation (28) with respect to *U*_DAQ_(*t*_*i*_) yields the equation system

33 $$\sum _{l=1}^{m}(\frac{\partial {a}_{l}}{\partial {U}_{{\rm{DAQ}}}({t}_{i})})\sum _{i=1}^{k}[\frac{\partial {U}_{{\rm{DAQ}}}({t}_{i})}{\partial {a}_{l}}\frac{\partial {U}_{{\rm{DAQ}}}({t}_{i})}{\partial {a}_{j}}-{r}_{i}\frac{{\partial }^{2}{U}_{{\rm{DAQ}}}({t}_{i})}{\partial {a}_{l}\partial {a}_{j}}]=-\,\frac{\partial {U}_{{\rm{DAQ}}}({t}_{i})}{\partial {a}_{j}},\,{\rm{with}}\,j=\mathrm{1,}\,\mathrm{2,}\,\mathrm{...,}\,m\mathrm{.}$$

This equation system is solved for the derivatives ∂*a*_*j*_/∂*U*_DAQ_(*t*_*i*_) for 1 ≤ *j* ≤ *m* and 1 ≤ *i* ≤ *k*, which allow for the calculation of the instrument function induced uncertainty ${\sigma }_{{a}_{j},{\rm{if}}}$ in each parameter *a*_*j*_:

34 $${\sigma }_{{a}_{j},{\rm{if}}}^{2}=\sum _{i=1}^{k}{(\frac{\partial {a}_{l}}{\partial {U}_{{\rm{DAQ}}}({t}_{i})}{\sigma }_{{\rm{U}},{\rm{if}},i})}^{2}\mathrm{.}$$

Now all three uncertainty components are known. The final uncertainty ${\sigma }_{{a}_{j}}$ in parameter *a*_*j*_ results from the noise induced uncertainty ${\sigma }_{{a}_{j},noise}$, the wavenumber axis induced uncertainty ${\sigma }_{{a}_{j},D}$ and the instrument function induced uncertainty ${\sigma }_{{a}_{j},{\rm{if}}}$ as

35 $${\sigma }_{{a}_{j}}^{2}={\sigma }_{{a}_{j},noise}^{2}+{\sigma }_{{a}_{j},D}^{2}+{\sigma }_{{a}_{j},{\rm{if}}}^{2}\mathrm{.}$$

### Data availability

All experimental datasets and evaluation results of this contribution are available on request from the authors.

## Experimental Validation

The theoretical derivation of the presented methods need to be applied to real measurement data demonstration and validation. The first step is therefore the determination of an instrument function from 20 separate measurements of methane. These measurements are carried out using the same setup as for the latter actual measurements, without the need for additional equipment. After that, ten measurements of methane at ambient pressure are evaluated. The low pressure ensures that the line shapes stay narrow. This way diode ringing and instrumental broadening have an significant effect on the measured line shapes and thus need to be reproduced by the instrument function. The ambient pressure measurements are therefore mainly used as a test bench for the instrument function. After that ten measurements at a higher pressure of 8.732 bar are evaluated. These measurements were conducted with a methane mole fraction of 0.223. In combination with the elevated pressure this leads to some wavenumber intervals being dominated by total absorption. This way two of the claims made for the presented methods can be tested: evaluation at higher pressures and evaluation in case of partial total absorption. All measurements were conducted at room temperatures as this is the simplest way to achieve homogeneous temperatures in the absorption path.

### Experimental setup

**Figure 4:**
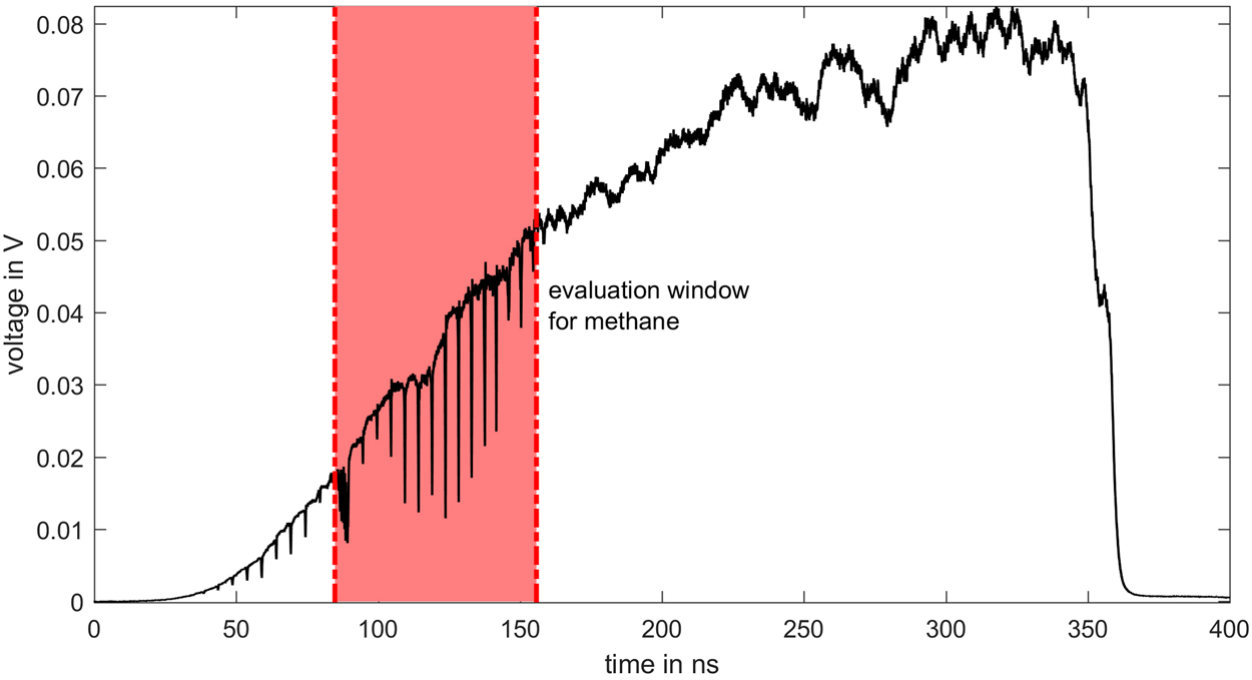
Example for the averaged raw data recorded by the oscilloscope. The measurement was performed in a methane air mixture at an absolute pressure of 0.980 bar, a methane mole fraction of 18.1 %, a temperature of 295.6 K and an absorption path length of 367 mm.

The spectrometer setup is equal to the one described and employed by Blume *et al*.^11^ and therefore only the most relevant parts are discussed here. The photo diode is a Newport Newfocus 1544-B-50 with a bandwidth of 12 GHz. The pulse response function of the photo diode is supplied by the manufacturer. For the data acquisition a LeCroy SDA 820Zi-A oscilloscope with a bandwidth of 20 GHz at a sampling rate of 40 GSps is used. The oscilloscope is also employed to directly average over 9849 consecutive SCL pulses. An example for the captured measurement is depicted in Fig. 4.

The measured methane is contained in a high pressure double-pass absorption cell with a total absorption path length of 367 mm. The reference temperature is measured with a thermocouple of type E in the cell. For room temperature measurements as they are conducted in this paper the combined reference temperature uncertainty of thermocouple and measurement system is ±2.9 K. The reference pressure is measured with a Keller PAA-33X 0–10 bar absolute pressure transducer with a measurement uncertainty of 5 mbar. To achieve well-defined methane-air mixtures the gases were premixed in a larger mixing vessel, before filling the evacuated measurement cell. This way the impact of insufficient mixing of the gases in the tubing is minimized. The reference values for the mole fraction were determined according to the pressure values in the mixing vessel. Therefore the reference uncertainty of the mole fraction is dependent on the absolute pressure and is given separately for each measurement.

### Instrument function

For the determination of the instrument function, measurements with high intensity gradients and therefore narrow absorption lines are needed to be able to resolve the caracteristic behavior of the high bandwidth photo diode. Hence measurements of pure methane at two pressure levels of 0.096 bar (abs.) and 0.497 bar (abs.) at a temperature of 295.5 K are used. Please note that the spectral resolution of the instrument is in the order of 0.1 *cm*^−1^ ^11^. Thus Dicke-Narrowing can be neglected in this investigation. The noise induced uncertainty of the determined instrument function decreases with the number of used measurements. For this evaluation a number of 10 measurements per pressure level in the wavelength region from 5995 cm^−1^ to 6145 cm^−1^ are used. For this wavenumber region the time-wavenumber-correlation *D*(*t*) is determined by applying the method described by Blume *et al*.^11^. As temperature, pressure and mole fraction are known, only the background *I*_0_ has to be determined, to be able to simulate a corresponding theoretical spectrum. This background is determined by fitting the whole model *U*_DAQ_ (Equation (16)) with variable background spline support points but fixed temperature, pressure and mole fraction to the measurements. The maximum number of spline support points is determined according to the maximum expected absorption line width *ν*_max_ as explained in section 2.2. For a mole fraction of 1, a temperature of 295.5 K and a pressure of 0.497 bar, Δ*ν*_max_ is 0.1473 cm^−1^. As the regarded wavenumber range spans 150 cm^−1^, Δ*ν*_max_ allows 1019 spline support points at maximum. Such a high number of support points would lead to a huge computational effort and additionally this estimation neglects the instrumental broadening of the line shape. Thus 80 equidistant spline support points are used for the background model as this allows for a sufficient description of the SCL background fluctuations.

**Figure 5:**
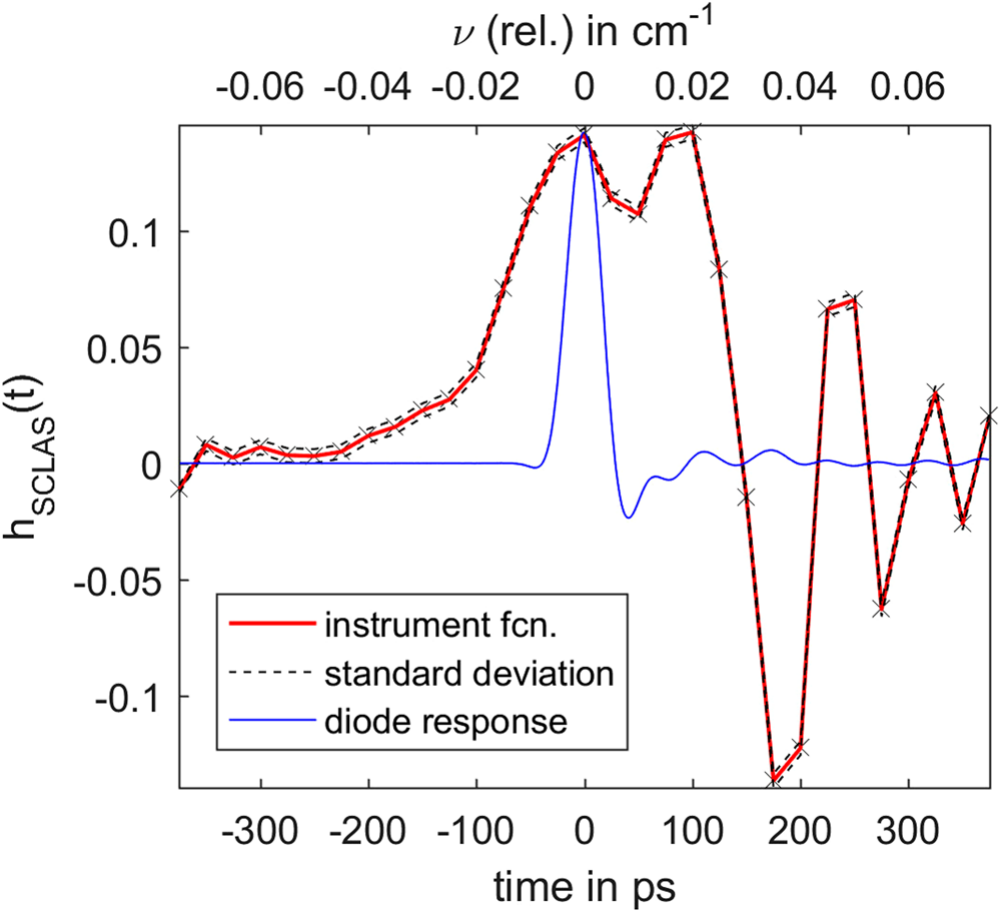
Instrument function determined from measurements. The instrument function was determined using SCLAS measurements with known absorber mole fraction, temperature and pressure and simulated signals. The diode response function of the Newport 1544-B-50 was supplied by the manufacturer and is normalized to the same peak height as the SCLAS instrument function for better visibility. The relative wavenumber axis is valid for the spectral region from 5995 cm^−1^ to 6145 cm^−1^.

If this fit was processed without assuming any instrument function the background could easily get distorted in the neighborhood of narrow absorption lines. Hence the yet unknown instrument function is roughly estimated as a Gauss pulse with a FWHM of 170 ps during the fit. With the resulting background *I*_0_ the simulated signal *I*_0_(*t*)e^−*A*(*D*(*t*),*T*,*p*,*q*)^ is known and the linear equation system (19) can be solved in the least square sense. The optimal number of non zero points in the instrument function was empirically determined to be 31, which is eqivalent to *μ* = 15. The resulting instrument function and its standard uncertainty are depicted in Fig. 5. The uncertainty of the instrument function was determined with the standard approach for linear least squares (see^21^). For an easier comparison the normalized diode pulse response is shown in the same figure.

As expected the determined instrument function shows a broadened pulse with a diode ringing artefact. The diode ringing does not exactly match that of the manufacturer supplied diode transfer function. This can be explained by the different input capacitances and impedances of the oscilloscopes used by the manufacturer and in this work. The high speed photo diode amplifier system is very sensitive to these changes. Another important difference between the diode response itself and the determined instrument function is the width of the actual pulse. The increased width, compared to the diode response, is introduced by the duration of the SCL pulse. Accordingly the small dip on top of the pulse represents another diode ringing artefact similar to the response on a step function.

Thus the effects observable in the instrument function can qualitatively be explained. The suitability of the instrument function for measurement evaluation is discussed in section 4.

### Simulated measurements

As already mentioned the current work aims to develop and validate the evaluation algorithms while the problem of accurate line data will be approached in future studies. In order to be able to differentiate between effects induced by the evaluation algorithms and effects induced by the line data, simulated measurement signals are evaluated. These simulated measurement signals build on the same absorption line data as the evaluation. Thus the corresponding evaluation results can be regarded as unbiased by inaccurate line data. The simulation procedure is similar to the signal model described in the methods section. The only difference lies in the simulation of the initial intensity distribution. The real initial intensity distribution does not necessarily follow the restrictions of the spline curve assumed in the model. Hence for the simulated signals differently scaled and filtered random signals are summed in order to generate a background distribution similar to a real signal. After applying the instrument function on the simulated signals, Gaussian white noise is applied to them in order to achieve different signal to noise ratios (SNR). Here SNR is defined as the maximum difference between a signal and the corresponding background without absorption divided by the standard deviation of the noise:

36 $${\rm{SNR}}=\,{\rm{\max }}({U}_{\mathrm{DAQ},{\rm{without}}{\rm{absorption}}}-{U}_{\mathrm{DAQ},{\rm{with}}{\rm{absorption}}})/{\sigma }_{{\rm{noise}}}$$

SNRs of 20, 100 and infinity, which corresponds to zero noise, were simulated for both gas parameter sets (ambient and high pressure). For each SNR and pressure level 100 simulated signals were evaluated. The mean and standard deviation of each evaluation series are summarized in Table 1. Similar to the evaluation of the experimental data in sections 3.4 and 3.5 the background model consists of 45 equidistant spline support points. Further explanation of this choice is given in section 3.4. As expected the standard deviations in all parameters increase with decreasing SNR. The simulations without noise (SNR = ∞) still show small deviations in the mean value. This is because the spline background model cannot fully represent the random background used in the signal simulation. Nonetheless the resultant mean values do not show any relevant accuracy problems of the evaluation procedure. Thus if accuracy problems are detected in real measurement evaluation the cause of these problems can be devoted either to the line data or the instrument function.

**Table 1:** Evaluation results of simulated signals for ambient and high pressure.

SNR	*T* in K (*σ*_*T*_ in K)	*p* in bar (*σ*_*T*_ in mbar)	*q* (*σ*_*q*_ in ppm)
SNR	$\hat{T}=295.6\,{\rm{K}}$	$\hat{p}=0.980\,{\rm{bar}}$	$\hat{q}=0.1806$
Inf	295.9 (0.00)	0.977 (0.0)	0.1812 (2)
100	295.8 (0.72)	0.978 (8.3)	0.1810 (1484)
20	295.6 (3.74)	0.978 (44.4)	0.1811 (7775)
SNR	$\hat{T}=295.6\,{\rm{K}}$	$\hat{p}=8.732\,{\rm{bar}}$	$\hat{q}=0.2235$
Inf	295.6 (0.00)	8.725 (0.1)	0.2236 (2)
100	295.6 (0.61)	8.724 (42.3)	0.2237 (875)
20	295.3 (2.43)	8.699 (220.8)	0.2241 (4349)

The signals were simulated for three different SNR values. Per pressure case and SNR value a series of ten measurements were evaluated. The table shows the mean value and standard deviation of each series.

### Ambient pressure measurements

**Figure 6:**
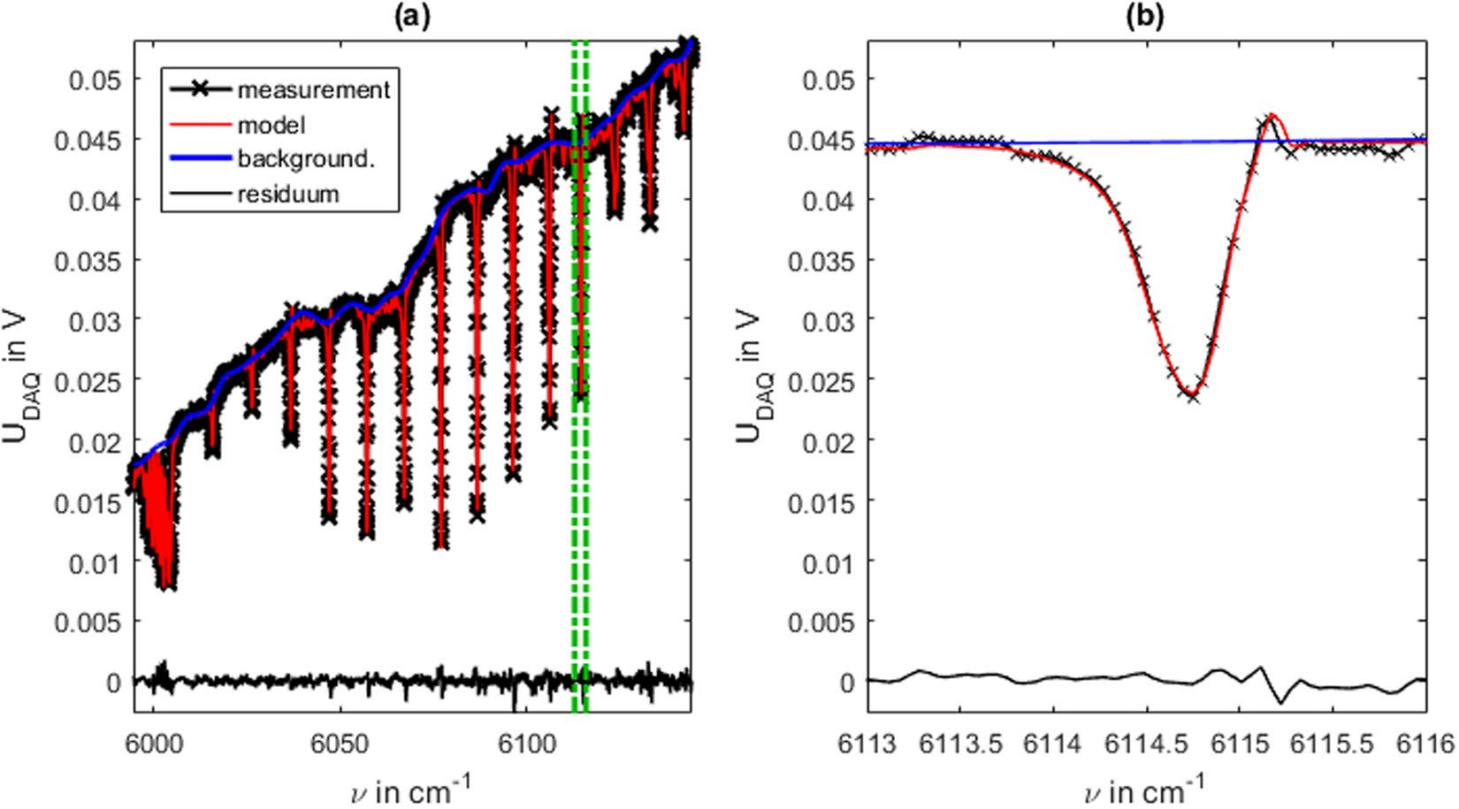
Evaluation of an SCLAS measurement at ambient pressure. Measurement of a methane-air-mixture at 0.980 bar, 295.6 K and an methane mole fraction of 18.06% (reference measurement). (**a**) measurement signal, fitted model and fit residuum in the evaluated spectral region. (**b**) detail view of the signal part marked in (**a**).

The experimentally determined instrument function is now applied in the evaluation of methane-air-mixtures. To validate the quality of the instrument function ten ambient pressure measurements at 0.980 bar, 295.6 K and a methane mole fraction of 18.06% are evaluated. The ambient pressure ensures that the diode-ringing effects are still visible and therefore have to be described by the instrument function in order to produce reasonable results. As the actual pressure is usually not known beforehand, a maximum pressure of 10 bar is assumed in order to determine the number of background spline support points. For the methane absorption lines in the regarded spectral region this leads to a maximum FWHM of Δ*ν*_max_ = 2.96 cm^−1^. Thus with the spectral range spanning 150 cm^−1^ the maximum allowable number of equidistant background spline support points is 51. As this estimation is just a rule of thumb and does not regard effects like superimposing absorption lines, the number of spline support points is chosen conservatively to be 45. Thus temperature, pressure and mole fraction as well as 45 equidistant background spline support points are fitted to the measurement data during the evaluation. The fitting result of one of the ten measurements is shown in Fig. 6. The detail view in Fig. 6b shows a very good agreement in the absorption line shape. The diode ringing is also visible in the right line shape wing with its steep incline and oscillation. While a small deviation in the ringing overshoot is still visible, no strong impact on accuracy is to be expected as the actual line shape and broadening are sufficiently reproduced.

The resulting values for temperature, pressure and mole fraction for the ten measurements are given in Table 2. The table also includes the uncertainties of the values (neglecting HITRAN uncertainties).

**Table 2:** Evaluation results of ten methane measurements at ambient pressure.

Measurement	1	2	3	4	5	6	7	8	9	10	mean	STD
$\hat{T}$ in K	295.6	295.6	295.6	295.6	295.6	295.6	295.6	295.6	295.6	295.6	304.4	1.16
*T* in K	305.8	305.8	304.7	303.7	303.5	302.1	305.5	304.1	304.8	304.3		
*σ*_*T*_ in K	2.91	3.60	3.31	3.37	3.04	2.98	3.17	3.12	3.28	3.36		
*σ*_*T*,*noise*_ in K	0.00	0.00	0.00	0.00	0.00	0.00	0.00	0.00	0.00	0.00		
*σ*_*T*,*D*0_ in K	2.45	3.21	2.91	2.98	2.61	2.54	2.75	2.70	2.88	2.97		
*σ*_*T*,*if*_ in K	1.58	1.63	1.59	1.58	1.55	1.55	1.58	1.57	1.58	1.57		
$\hat{p}$ in mbar	980	980	980	980	980	980	980	980	980	980	982	17.0
*p* in mbar	989	1010	987	974	977	979	989	960	955	1002		
*σ*_*p*_ in mbar	75.0	94.4	86.3	89.1	79.5	78.1	85.0	80.3	81.3	90.1		
*σ*_*p*,*noise*_ in mbar	0.0	0.0	0.0	0.0	0.0	0.0	0.0	0.0	0.0	0.0		
*σ*_*p*,*D*0_ in mbar	74.3	93.5	85.5	88.2	78.7	77.3	84.1	79.6	80.5	89.3		
*σ*_*p*,*if*_ in mbar	10.6	13.3	12.0	12.4	11.0	11.0	11.7	11.1	11.2	12.5		
$\hat{q}$ in %	18.06	18.06	18.06	18.06	18.06	18.06	18.06	18.06	18.06	18.06	19.42	0.35
*q* in %	19.20	18.88	19.29	19.53	19.47	19.37	19.33	19.87	20.09	19.14		
*σ*_*q*_ in %	1.33	1.60	1.55	1.65	1.46	1.42	1.53	1.54	1.57	1.58		
*σ*_*q*,*noise*_ in %	0.16	0.18	0.18	0.18	0.17	0.16	0.17	0.18	0.18	0.18		
*σ*_*q*,*D*0_ in %	1.31	1.57	1.52	1.62	1.44	1.40	1.50	1.51	1.54	1.55		
*σ*_*q*,*if*_ in %	0.21	0.24	0.23	0.24	0.22	0.22	0.23	0.23	0.23	0.24		

The values $\hat{T}$, $\hat{p}$ and $\hat{q}$ denote the reference measurements. The value of *σ* gives the standard deviation of the corresponding variable and *σ*_noise_*, σ*_*D*0_ and *σ*_if_ accordingly describe the contribution of noise, wavenumber and instrument function uncertainty to *σ*. The column standard deviation(STD) shows the empirical standard deviation of the measurement series.

The low pressure SCLAS evaluations show maximum temperature deviations of 10.2 K (measurement 1 & 2) from the reference measurement of 295.6 ± 2.9 K. Nonetheless the temperatures determined by SCLAS only fluctuate in a range of 3.7 K with a mean value of 304.4 K. The calculated precision uncertainties give values between 2.91 K and 3.60 K. These uncertainties are mainly induced by wavenumber and instrument function uncertainty. As the temperature was constant during the ten low pressure measurements, the determined temperature values can be used to empirically estimate the precision uncertainty of the SCLAS evaluations using the empirical standard deviations over the ten measurements. Hereafter standard uncertainties estimated by the standard deviation of the measurement series are called empirically estimated uncertainties while the standard uncertainties calculated using the approach presented in this paper are referred to as directly calculated. For the temperature measurements this leads to an empirically determined standard deviation of 1.16 K. Therefore the directly calculated precision uncertainty gives greater values but in the same order of magnitude as the empirically estimated ones.

The pressure results in the ambient pressure measurements 1 to 10 show a maximum deviation of 0.030 bar from the reference measurement of 0.980 ± 0.005 bar. The directly calculated standard uncertainties overestimate the empirically estimated standard uncertainty of 0.017 bar but are still in the same order of magnitude. Regarding the mean value of 0.982 bar and the standard uncertainty no relevant inaccuracy can be identified.

The mole fraction results in the ambient pressure measurements show a maximum deviation of 2.3% from the reference measurement of 18.06 ± 0.5%. The directly calculated standard uncertainties overestimate the empirically estimated value of 0.35% by a factor of four. But similar to the derived temperature, the mean value of the mole fraction 19.42% deviates from the reference value of 18.06%. This behaviour is discussed in section 4. Regarding the direct uncertainty estimation the uncertainties in all parameters are overestimated to a reasonable degree, which can mainly be attributed to the wavenumber induced uncertainty.

### High pressure measurements

Additionally to the ten ambient pressure measurements, ten high pressure measurements at 8.732 bar and 295.6 K of a methane-air-mixture are evaluated. The methane mole fraction of 22.3% in combination with the elevated pressure leads to spectral regions with total absorption. As the pressure is not assumed to be known beforehand, a maximum pressure of 10 bar is assumed. Thus similar to the low pressure case temperature, pressure, mole fraction and 45 background spline support points are fitted to the measurement data.

**Figure 7:**
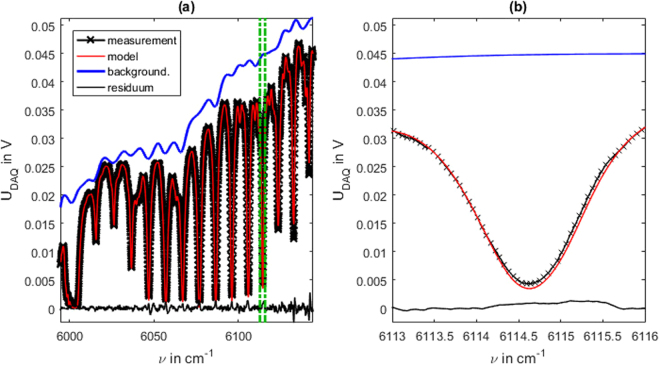
Evaluation of an SCLAS measurement at elevated pressure. Measurement of a methane-air-mixture at 8.732 bar, 295.6 K and an methane mole fraction of 22.3 % (reference measurement). (**a**) Measurement signal, fitted model and fit residuum in the evaluated spectral region. (**b**) Detail view of the signal part marked in (**a**).

The fitting result of one of these high pressure measurements is shown in Fig. 7. The diode ringing is not visible because of the strong collisional broadening.

As visible in the detail view in Fig. 7 the model line shapes slightly deviate from measured shapes. The probable reason for this are inaccuracies in the line data for collisional broadening and pressure induced line shift. Compared to the low pressure measurements the fluctuations in the fitted background are significantly stronger. These fluctuations can be explained with the strong absorption in these regions. As the received intensity signal as well as the expected transmittance exp(−*A*) approach zero, (1) does not allow the determination of a definite *I*_0_. As a result the fitted background in regions with strong absorption is not trustworthy. Nonetheless, the important evaluation variables temperature, pressure and mole fraction are not affected by this. The derivatives of the signal model *U*_DAQ_ with respect to these variables in regions of total absorption, are small compared to regions with weaker absorption. Hence the influence of these variables on the cost function is very low in strong absorption regions. Thus the determined optimum of the cost function in these variables is not influenced by the total absorption region but by the wings of the absorption lines, that still show moderate absorption.

The resulting values for temperature, pressure and mole fraction are given in Table 3 with their corresponding uncertainties. The high pressure SCLAS evaluations (measurements 11 to 20) show maximum temperature deviations of 17.5 K (measurement 20) from the reference measurement of 295.6 ± 2.9 K. Obviously the deviation from the reference increased, while the fluctuation range of 1.0 K decreased compared to the low pressure measurements. The directly calculated precision uncertainties give values between 1.34 K and 2.22 K while the standard deviation over the ten measurements is 0.31 K. Thus the uncertainty is overestimated but still in the same order of magnitude.

**Table 3:** Evaluation results of ten methane measurements at high pressure.

Measurement	11	12	13	14	15	16	17	18	19	20	mean	STD
$\hat{T}$ in K	295.6	295.6	295.6	295.6	295.6	295.6	295.6	295.6	295.6	295.6	312.6	0.31
*T* in K	312.8	312.6	312.4	312.9	312.8	312.1	312.7	312.3	312.4	313.1		
*σ*_*T*_ in K	1.48	2.04	1.60	1.34	1.34	1.58	2.22	1.56	1.37	1.49		
*σ*_*T*,*noise*_ in K	0.00	0.00	0.00	0.00	0.00	0.00	0.00	0.00	0.00	0.00		
*σ*_*T*,*D*0_ in K	1.37	1.95	1.49	1.21	1.22	1.48	2.14	1.45	1.26	1.38		
*σ*_*T*,*if*_ in K	0.56	0.59	0.57	0.56	0.56	0.56	0.60	0.56	0.56	0.57		
$\hat{p}$ in mbar	8732	8732	8732	8732	8732	8732	8732	8732	8732	8732	8571	32.5
*p* in mbar	8604	8603	8534	8577	8563	8516	8590	8559	8551	8617		
*σ*_*p*_ in mbar	198.7	303.9	221.9	173.4	175.7	218.7	341.0	218.8	177.2	196.8		
*σ*_*p*,*noise*_ in mbar	0.0	0.0	0.0	0.0	0.0	0.0	0.0	0.0	0.0	0.0		
*σ*_*p*,*D*0_ in mbar	197.0	301.3	220.0	171.9	174.2	216.8	338.0	216.9	175.7	195.1		
*σ*_*p*,*if*_ in mbar	25.8	39.6	28.9	22.5	22.9	28.7	44.5	28.6	22.9	25.7		
$\hat{q}$ in %	22.35	22.35	22.35	22.35	22.35	22.35	22.35	22.35	22.35	22.35	24.47	0.07
*q* in %	24.38	24.43	24.47	24.49	24.55	24.59	24.40	24.46	24.50	24.39		
*σ*_*q*_ in %	0.38	0.56	0.43	0.34	0.35	0.43	0.63	0.42	0.35	0.38		
*σ*_*q*,*noise*_ in %	0.08	0.10	0.09	0.08	0.08	0.09	0.11	0.09	0.08	0.08		
*σ*_*q*,*D*0_ in %	0.36	0.55	0.41	0.33	0.33	0.41	0.61	0.40	0.33	0.36		
*σ*_*q*,*if*_ in %	0.06	0.08	0.07	0.06	0.06	0.07	0.09	0.07	0.06	0.06		

The values $\hat{T}$, $\hat{p}$ and $\hat{q}$ denote the reference measurements. The value of *σ* gives the standard deviation of the corresponding variable and *σ*_noise_*, σ*_*D*0_ and *σ*_if_ accordingly describe the contribution of noise, wavenumber and instrument function uncertainty to *σ*. The column standard deviation(STD) shows the empirical standard deviation of the measurement series.

The mean value of the pressure measurement in the high pressure case matches the reference pressure value to within 216 mbar. The direct calculation of the uncertainty overestimates the standard deviation of the measurement series, but still shares the same order of magnitude.

Similar to the temperature the mean value of the mole fraction deviates by 2.24% from the reference value and the uncertainties are overestimated but in the same order of magnitude.

Summarized the high pressure case shows an overestimation of temperature and mole fraction similar to the ambient pressure case. Additionally the uncertainties in all parameters are overestimated to a reasonable degree, which again can be attributed to the wavenumber induced uncertainty. The reasons for this are discussed in the next section.

## Discussion

Ambient pressure measurements as well as high pressure measurements show reasonably good agreement in the spectral line shape between model and measurement. Especially in the ambient pressure measurements the distortion of the line shapes and the diode-ringing is clearly visible and correctly depicted by the model.

Furthermore the pressures derived from the spectroscopic measurements match the reference pressure measurements very well. These observations indicate a good quality of the estimated instrument function.

As already mentioned the temperature as well as the mole fraction value show a deviation in their mean values from the reference values for both pressure levels. As it was shown in the evaluation of simulated signals the evaluation procedure gives accurate results if the line data and the instrument function match the real values. Because the derived temperature is mainly dependent on the ratio between the line strengths and the instrument function affects all absorption lines similarly, the more probable cause for the deviation in temperature is inaccurate line data. Additionally this deviation in the temperature can also explain the deviation observed for mole fractions, as the methane absorption line strengths in the regarded region decrease with increasing temperature. Therefore an underestimated temperature in the fitting optimum leads to the compensation of the reduced line strength by an increased value for the mole fraction. Thus the main reason for both deviations is most probably inaccurate line data. The pressure was determined accurately in both pressure levels. This could only be achieved by the novel determination method for the instrument function.

The results of the uncertainty estimation show for both pressure levels a reasonable overestimation of the empirical estimated standard deviations. This overestimation is mainly caused by the wavenumber axis uncertainty while the noise and instrument function induced uncertainty alone would give a tight estimate of the standard deviation. This can be explained as equation (27) is only a upper bound for the real trigger jitter which can be significantly smaller. Therefore in order to get a tighter estimate the real trigger jitter in the measurement system has to be measured. This demands a lot of effort in terms of equipment and setup as the data acquisition used here already has a very high acquisition rate that has to be exceeded in order to lower the bound on wavenumber axis uncertainty. On the other hand neglecting the wavenumber axis uncertainty is not an option as slight changes on the setup (e.g. longer trigger lines) can easily flatten the trigger edges and therefore introduce a not neglectable trigger jitter. Thus the presented detailed uncertainty estimation gives reasonably conservative estimates of the precision uncertainty from a single measurement. This capability is important in many experiments that can only be conducted a very limited number of times.

## Conclusion

The current work provides a set of algorithms that facilitates the simultaneous evaluation of SCLAS measurements in temperature, pressure and absorber mole fraction without recording an additional background measurement, also at elevated pressures. An important part of this evaluation is the *in-situ* determination of the spectrometer instrument function from low pressure measurements, without additional hardware. Only with this fully specified instrument function the exact evaluation of pressures becomes feasible. In addition a detailed statistical uncertainty propagation for several uncertainty sources and all evaluation parameters is proposed. This enables the estimation of precision uncertainties of all parameters from a single measurement.

These algorithms are applied to methane measurements with an absorption path length of 367 mm at ambient pressure (0.980 bar) and elevated pressures (8.732 bar). Especially the ambient measurements depict a very good agreement with the determined instrument function. The resulting temperature, pressure and mole fraction values match the reference measurements reasonably well for both cases. Furthermore the precision uncertainty calculation results give a conservative estimate of the fluctuations observed in both measurement cases. Thus the uncertainty propagation can be used to estimate the achievable precision of SCLAS measurements. The results promise precision uncertainties of below 3 K in temperature, below 50 mbar in pressure and below 0.4 % in methane mole fraction for the regarded experimental setup. This shows the high potential of SCLAS as a multi parameter line of sight measurement method.

Of course this potential can only be fully exploited with accurate line data. Therefore an important step to the maturity of SCLAS is the derivation of more accurate line data or other spectrum models. Additionally the simulation of absorbance spectra has to be optimized as the currently achievable evaluation time is limited by the high computational effort of simulating several thousand absorption transitions.

The presented uncertainty estimation is limited to the measurement uncertainty without line data uncertainty as it was only used as a measure of the achievable precision. By applying an analogous approach for the error propagation of the line data, the total uncertainty of SCLAS measurements can be directly estimated. With these enhancements SCLAS can be used as a valuable tool in research and development without the need for additional reference sensors.
